# Perceived protein needs and measured protein intake in collegiate male athletes: an observational study

**DOI:** 10.1186/1550-2783-8-9

**Published:** 2011-06-21

**Authors:** Elizabeth A Fox, Jennifer L McDaniel, Anthony P Breitbach, Edward P Weiss

**Affiliations:** 1 Department of Nutrition and Dietetics, Saint Louis University, 3437 Caroline St, Room 3076, Saint Louis, MO, 63104, USA; 2 Athletic Training Education Program, Saint Louis University, 3437 Caroline St, Room 2004, Saint Louis, MO, 63104, USA

## Abstract

**Background:**

Protein needs for athletes are likely higher than those for the general population. However, athletes may perceive their protein needs to be excessively high. The purpose of this research was to compare collegiate athletes' perceived protein needs and measured protein intake to the recommended protein intake (RDI) for healthy adults (i.e. 0.8 g/kg/d) and to the maximum beneficial level for strength-trained athletes (i.e. 2.0 g/kg/day).

**Methods:**

Perceived protein needs were quantified in 42 strength-trained collegiate male athletes by using a survey that asked the athletes to provide their perception about protein needs in specific quantitative terms (i.e. g/kg/d). Perceived protein needs were also determined by having the athletes select a daylong menu that they perceived to have adequate protein content from a collection of 5 isoenergetic menus, which differed in terms of protein content. Actual protein intake was quantified using 3-day food records and nutrient analysis. Single sample t-tests were used to compare protein intake and perceived protein needs to 0.8 g/kg/day and 2.0 g/kg/day.

**Results:**

When asked to provide, in quantitative terms, protein needs for athletes, 67% of the athletes indicated "do not know." Of the remaining 33% of athletes, all gave values greater than 2.0 g/kg/d (mean 21.5 ± 11.2 g/kg/d, p = 0.14 vs. 2.0 g/kg/d). Based on the menu selection method for determining perceived protein needs, the athletes indicated that their protein needs were 2.4 ± 0.2 g/kg/d, which was greater than the RDI for protein (p < 0.0001) and tended to be greater than the maximally beneficial protein intake of 2.0 g/kg/d (p = 0.13). Measured protein intake was 2.0 ± 0.1 g/kg/d, which was greater than the RDI (p < 0.0001) but not different from the maximally beneficial protein intake of 2.0 g/kg/d (p = 0.84).

**Conclusions:**

Male collegiate athletes recognize that their protein needs are higher than that of the general population and consume significantly more protein than recommended in the RDI. However, it also appears that athletes are not aware of objective recommendations for protein intake and may perceive their needs to be excessively high. This study highlights the need for nutrition education in collegiate athletes, in particular nutrition education on macronutrient distribution and protein needs.

## Background

Dietary protein intake and protein supplementation are routinely excessive among athletes. Even the typical American diet generally exceeds the 0.8 g/kg/d reference daily intake (RDI) for protein. According to NHANES 2003-2004, adults aged 19-30 yr have protein intakes in the range of 1.0-1.5 g/kg/d [1]. Two studies have evaluated the dietary practices of national collegiate division I football players. Cole et al. quantified intake using a 3-day diet record and found that the football players had lower intake of calories, carbohydrate and fat, but more protein when compared to age- and sex-matched subjects from NHANES III [2]. Another study evaluated intake using a self-administered nutrition-screening questionnaire that focused on dietary practices and attitudes. They found that 42% of the football players took (protein or other) supplements, with the most popular being creatine (36%). They also found that greater than 50% of the subjects in the study had the improper perception that protein was the primary source of energy for muscle [3].

It is generally accepted that athletes have increased protein needs. Although there is no special RDI for protein intake among athletes, the position statement of the International Society of Sports Nutrition states that exercising individuals' protein needs are between 1.4 and 2.0 g/kg/day, depending upon mode and intensity of exercise, quality of protein, and status of total calorie and carbohydrate intake [4]. Protein intakes greater than this do not provide benefits [2]. For example, one study found that dietary protein intakes of 2.6 g/kg/day during resistance-exercise training in young males did not result in larger increases in strength or body mass beyond those that occurred with a protein intake of 1.35 g/kg/day [5]. Furthermore, protein intakes of 2.8 g/kg/d did not result in greater muscle protein synthesis rates, as compared to 1.8 g/kg/d [6].

Adding to the confusion among athletes and coaches about protein needs is the extensive and influential marketing by protein supplement companies. Furthermore, it is attractive to rationalize that muscle is largely made up of protein and therefore, high protein intake must be important for large muscles. Collectively, all of these factors might contribute to the perception among athletes that protein needs are very high, which could result in excessive use of protein supplements and excessive dietary protein intakes. The purpose of the present study was to investigate collegiate athletes' perceived protein needs and actual protein intake and to compare these findings with 0.8 g/kg/day as the RDI for protein in healthy adults and the maximum beneficial level for athletes of 2.0 g/kg/day.

## Methods

### Subjects

NCAA Division I collegiate male athletes actively engaged in strength and endurance training were recruited for this study from Saint Louis University. Subjects were excluded if they were not between the ages of 18-35 yr, were not participating in a collegiate sport at the time of the study, or were diagnosed with a medical condition that required them to follow a special diet, including celiac disease, diabetes and irritable bowel disease (IBD). Strength-trained athletes were considered to be any athletes who performed strength/power lifting ≥ 3 days per week with a duration of ≥30 minutes per session. Forty-two Saint Louis University male athletes that met the inclusion criteria participated in this study. All provided informed written consent to participate in the study, which was approved by the Saint Louis University Institutional Review Board. All data were coded and protected to meet the standards for confidentiality for all subjects.

### Study Design

This was an observational study in which the measured protein intake and perceived protein needs were evaluated and compared to the RDI for protein intake and to the maximum beneficial level of protein intake for athletes.

### Subject Characteristics

Height, weight and age were self-reported. Body mass index (BMI) was calculated from height and weight in kg/m^2^.

### Body Composition

Chest, abdomen, and thigh skinfold thicknesses were measured with a Lange callipers by using standard methodology as published elsewhere [7]. Each site was measured 3 times or more until 3 measures at a given site were within 0.1 mm. The Jackson and Pollock 3-site equation was used to calculate body density. The Brozek equation was used to calculate lean body mass (LBM) and percentage body fat [7].

### Perceived Protein Needs

Subjects were asked to complete a protein survey and a protein menu selection to assess perceived protein needs. The protein survey was used to identify the athletes' perception of protein needs by asking the subjects to list, in g/kg/d, g/lb/d and % daily calories, "how much protein do you think you need to get the biggest benefit from your training program and to get the best performance in your sport?" Subjects were presented with the option of selecting "do not know". The survey also assessed subjects' seasonal changes in protein intake and frequency, intensity, type and time for endurance and strength-trained activities using self-reported answers including the Borg Scale for rating of perceived exertion.

It was anticipated that many athletes would not be able to report a specific value for protein intake (i.e. g/kg/d or % total energy intake) to reflect their perceptions about protein needs. However, it seemed likely that most would be able to look at a menu of specific food items and indicate if they believed that the menu had adequate protein to meet their needs. Therefore, subjects were asked to review 5 menus that represented isoenergetic diets but varied in terms of protein levels (0.8 g/kg/d, 1.42 g/kg/d, 2.0 g/kg/d, 4.0 g/kg/d, 5.0-6.0 g/kg/d). Subjects were blinded to the actual amount of protein. Each of the protein menus only listed specific foods and their serving sizes and provided the option to add in a protein supplement. Menu sets were available at 3 calorie levels (3100 kcal/d, 3500 kcal/d, 3800 kcal/d). Each subject received the menu set that corresponded most closely to their estimated energy needs, as estimated using published equations [8]. The subjects were instructed to select one of the 5 menus that they perceived would meet their protein needs during their highest level of training. From the protein content of the selected menu and the subjects' body weight, perceived protein needs were calculated as g/kg/d. For those subjects who chose to add an additional protein supplement to a selected menu, the supplemental protein was included in the calculation of perceived protein needs.

### Measured Protein Intake

Actual protein intake was determined by using 3-day food records and nutrient analysis. Subjects received 3-day food record instruction and education on accurate portion size estimation by a Registered Dietitian (RD). Subjects completed the food record by recording all foods and beverages consumed on two week days and one weekend day. For the follow up visit, subjects met with the same RD and reviewed the 3-day food records to clarify any questions/concerns on portion sizes or food items. Food records were analyzed by the study RD using Food Processor SQL Nutrition & Fitness software (10.6.0, ESHA Research, Salem, Oregon).

### Statistical Analyses

Single sample t-tests were used to compare measured protein intake and perceived protein intake to recommended intakes of 0.8 g/kg/day and 2.0 g/kg/day. A paired t-test was used to compare perceived protein needs from the menu selection to actual protein intake. Data analysis was completed using PASW Statistics 18 software (SPSS Inc., Chicago, IL) and the significance level was set at p ≤ 0.05. Data are presented as means ± standard error unless otherwise noted.

## Results

### Subject Characteristics

Subjects included men's basketball (n = 14) and baseball players (n = 28) (Table 1). Mean body fat percentage was in the acceptable range for male athletes and subjects' BMI averaged in the high end of normal, as expected with lean athletes. Strength exercise frequency (mean ± SD) was 4.0 ± 1.1 days per week, for 2.3 ± 1.4 hours per day at an average intensity of 7.3 ± 1.4, using the 1-10 Borg scale for rating of perceived exertion.

**Table 1:** Subject Characteristics

Age (yrs)	19.7 ± 1.2
Height (cm)	188.0 ± 8.2
Weight (kg)	88.0 ± 11.1
BMI (kg/m^2^)	24.8 ± 2.2
LBM (kg)	78.7 ± 8.7
Body Fat %	10.4 ± 3.1
Energy intake (calories)	3648 ± 1170
% Calories from Carbohydrate	46.4 ± 8.6
% Calories from Fat	33.2 ± 7.6

Body mass index (BMI), lean body mass (LBM). Data are presented as means ± standard deviation. N = 42

### Perceived Protein Needs

The results of the protein survey showed that 67% of the athletes selected "do not know" when asked to provide the protein recommendations for athletes in terms of g/kg/d, g/lb/d, or percentage of total calories. The remaining 33% of the athletes indicated that the mean recommended protein intake for athletes was 21.5 ± 11.2 g/kg/d (p = 0.14 vs. 2.0 g/kg/d) or 27 ± 3% of total energy intake. One subject reported the mean recommended protein intake as 200 g/kg/d (i.e. 250-fold greater than the RDI). When this subject was excluded, the mean recommended protein intake reported was 8.7 ± 4.1 g/kg/d. When comparing these numbers to the RDI for protein of 0.8 g/kg/day (p = 0.05), the maximum beneficial level of 2.0 g/kg/day (p = 0.10), it is apparent that these athletes not only perceive their protein needs to be much greater than current recommendations, but also are not aware of what the current recommendations are for protein intake in strength-trained athletes.

**Figure 1:**
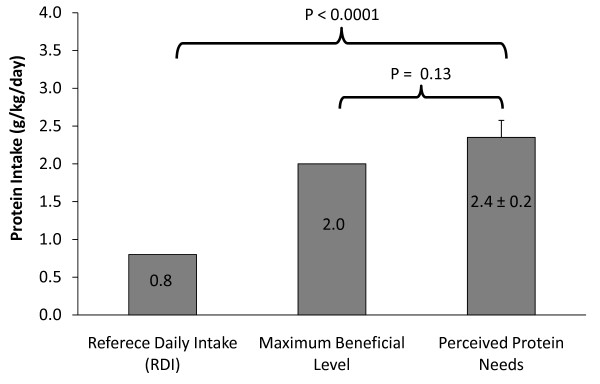
**Perceived Protein Needs**. The recommended protein intake (RDI), maximum beneficial level of protein intake, and the mean perceived protein needs, as determined by protein menu selection, in grams of protein per kg of body weight per day.

Results from the menu selection method for quantifying perceived protein needs showed that 31% of the athletes selected the menu corresponding to the protein RDI of 0.8 g/kg/d, 31% selected the menu corresponding to 1.4 g/kg/d, 12% selected 2.0 g/kg/d, 21% selected 4.0 g/kg/d and < 1% selected 5.0-6.0 g/kg/d. In addition, 33% of the athletes chose to add a protein supplement to the menu, with the mean daily dosage of 45 grams. The mean perceived protein needs from the menu selection was 2.4 ± 0.2 g/kg/d (Figure 1), which was significantly greater than the RDI of 0.8 g/kg/day (p < 0.0001). Although this value is ~20% greater than the maximum beneficial level for protein intake in athletes of 2.0 g/kg/day, it was not statistically different from 2.0 g/kg/d (p = 0.13).

### Actual Macronutrient and Energy Intake

**Figure 2:**
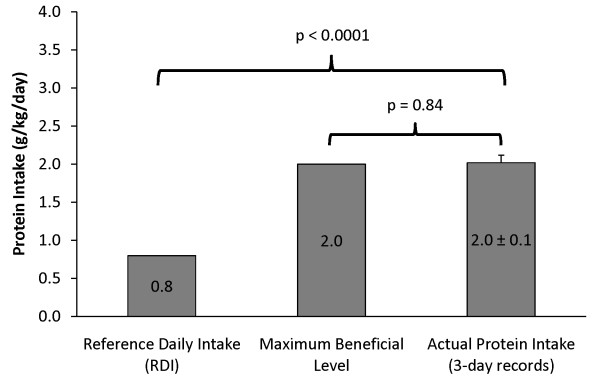
**Actual Protein Intake**. The RDI, maximum beneficial level of protein intake, and the mean actual protein intake as determined by 3-day food record analysis in grams of protein per kg of body weight per day.

Based on 3-day food records, mean protein intake was 173 ± 7 grams per day, or 2.0 ± 0.1 g/kg/d. This was significantly higher (p < 0.0001) than the RDI of 0.8 g/kg/d for healthy adults (Figure 2). However, protein intake was not significantly different from the maximum beneficial level of protein intake of 2.0 g/kg/d (p = 0. 84) or from perceived protein needs as determined by menu selection (p = 0.16). The protein survey showed that 76% of the athletes used protein supplements, with a mean daily intake of 46 ± 8 grams.

**Figure 3:**
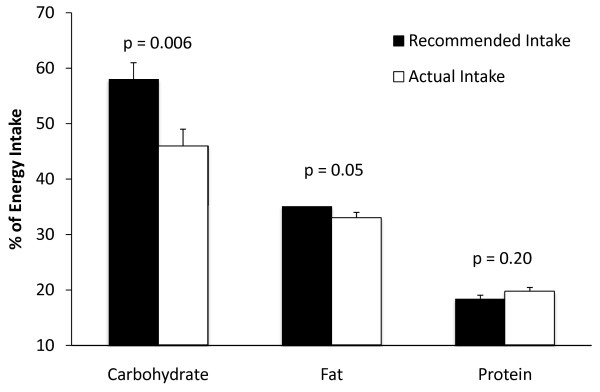
**Recommended vs. Actual Macronutrient Intake**. The recommended macronutrient distribution for athletes compared to measured macronutrient intakes. Recommended carbohydrate intake was calculated as a percentage of total energy intake based on the minimum recommended carbohydrate intake for athletes (i.e. 6 g/kg/d) [9], body weight, and total energy intake. The upper limit for fat intake was set at 35% based on recommendations [9]. Recommended protein intake was calculated as a percentage of total energy intake based on the upper end of the recommended range for protein intake athletes (i.e. 1.7 g/kg/d) [9], body weight, and total energy intake.

The average daily energy intake, as estimated by analysis of 3-day food records, was 3648 ± 173 kilocalories, with an average of 46 ± 2% of those calories coming from carbohydrate, 33 ± 1% from fat, and 21 ± 1% from protein. Although the intakes of fat and protein were not significantly different from recommended intakes for athletes [9], carbohydrate intake was lower than the recommended levels (Figure 3).

## Discussion

Results from this study show that in male collegiate athletes, perceived protein needs were significantly greater than the RDI for protein, but not significantly different than the 2.0 g/kg/day maximum beneficial level for training and physical performance. It was not surprising that the subjects perceived needs were significantly greater than the 0.8 g/kg/day RDI, considering the extensive marketing of protein supplements to athletes and the protein focused culture of strength coaches and athletes. Furthermore, the most recent literature review on protein requirements in strength-trained athletes concludes that protein requirements for these individuals are elevated due to: 1) enhanced oxidation rates of endogenous amino acids during exercise, 2) the need for increased substrate to repair damaged muscle tissue, and 3) the capacity to maintain elevated protein synthesis for greater amounts of muscle tissue [10]. However, the level of unawareness among the athletes was surprising when they were asked to report current protein recommendations for strength-trained athletes; none of the subjects answered correctly and most selected the "do not know" response. When asked to indicate perceived protein needs by selecting a menu that would meet their protein needs during their highest level of training, the athletes on average identified menus providing 2.4 ± 0.2 g/kg/day, which is 3-fold greater then the RDI for protein. Furthermore, based on menu selection, more than 1 out of 5 athletes believed that their protein needs are ≥4 g/kg/d. Taken together, these findings indicate that collegiate athletes understand that their protein needs are greater than the RDI. However, they also indicate that many athletes perceive their protein needs to be above the maximum beneficial level of protein for training and athletic performance.

Similar to what was found for perceived protein needs, actual protein intake (2.0 ± 0.1 g/kg/d) was significantly greater than the RDI for protein, but not significantly different from the 2.0 g/kg/day maximum beneficial level for protein intake. Actual protein intake was comparable to perceived protein needs (p = 0.16) and to the 2.0 g/kg/day maximum beneficial level for protein intake in athletes. Food record analysis showed modest inappropriate macronutrient balance. Figure 3 compares actual macronutrient intake to the recommended macronutrient distribution for athletes [9]. Measured carbohydrate intake (% of total calories) was significantly less than (p = 0.006) the lowest recommended level and fat and protein intakes were near the highest recommended levels (p = 0.05 and p = 0.20, respectively). Taken together, high-normal fat and protein intakes resulted in suboptimal carbohydrate intake. Ultimately, this could increase the risk of glycogen depletion and performance impairment during training/competition, especially with repeated bouts of intense endurance exercise over a relatively short time period (i.e. < 24 hr).

In this study, there were limitations. Inaccurate estimation of portion sizes for food records may have lead to incorrect reporting of dietary intake; it is also possible that the subjects altered their dietary habits during the food diary recording period. To minimize these effects, the study RD provided and reviewed with subjects a food portion estimation handout prior to the 3-day food recording period and advised the subjects to avoid altering their usual diet. After the food diary was recorded, the RD reviewed the food records individually with each subject to clarify ambiguities before nutrient analysis was performed. Another limitation of this study is that we cannot determine why the subjects' protein intake was high. It is possible that the athlete's high protein intake is attributable to their own nutrition knowledge; alternatively, it may be largely due to influences from coaches and/or other athletes. In light of this limitation, our findings may not be applicable to athletes in other environments.

Excess protein intake (> 2.0 g/kg/d) likely has no beneficial effect on performance or training adaptations. For example, protein intakes of 2.6 and 2.8 g/kg/d do not provide benefits above and beyond those from intakes of 1.35, 1.4 and 1.8 g/kg/d, respectively [5,6,11]. Furthermore, even intakes of 2.0 g/kg/d may be excessive for this population of well trained athletes [9], as the highest protein needs are thought to occur in untrained individuals who are initiating training programs and undergoing net accrual of protein for tissue synthesis [12].

In contrast to the relatively well-known effects of protein intake on training adaptations and physical performance, little is known about the effects of a high-protein intake (i.e. intake well above the 0.8 g/kg/d RDI) on health-related outcomes. Research has consistently shown positive associations between higher dietary protein intakes and increased circulating concentrations of insulin-like growth factor 1 (IGF-1) [13,14]. Elevated IGF-1 levels may be associated with protection against age-related cognitive decline [15], cardiovascular disease [16] and osteoporosis [17]. However, IGF-1 appears to also promote carcinogenesis [18-21], as it promotes cell differentiation and proliferation and inhibits apoptosis [22]. Furthermore, inhibition of IGF-1 activity/signalling through pharmaceutical intervention or as a result of high levels of IGF binding protein may be associated with more favorable responses to chemotherapy, providing additional evidence for a potential role of IGF-1 in carcinogenesis [23,24]. In this context, and is the case for most nutrients, it may be prudent to consider that there may be an optimum for protein intake and that low intakes and high intakes may both be harmful. However, because there is a paucity of research on the health effects (or lack thereof) of chronically high protein intake, we do not know if protein intakes such as those seen in the athletes in our study would be expected to have adverse effects on health.

## Conclusions

Perceived protein needs and actual protein intake in male collegiate athletes are greater than the RDI for protein of 0.8 g/kg/d for healthy adults and greater than or equal to the maximum beneficial level for protein intake of 2.0 g/kg/d. These findings were accompanied by a modest inadequacy in carbohydrate intake, which could have implications for physical performance. Therefore, this study highlights the need for nutrition education in collegiate athletes, in particular nutrition education on macronutrient distribution and protein needs.

## Competing interests

The authors declare that they have no competing interests.

## Authors' contributions

EAF and EPW conceived the study idea and analyzed the data. EAF, EPW, and JLM designed the study. EAF carried out data collection, and drafted the manuscript. All authors contributed to the interpretation of results, critically reviewed the manuscript for intellectual content, and gave approval of the final version of the manuscript to be published.
